# P2Y_1 _receptor switches to neurons from glia in juvenile versus neonatal rat cerebellar cortex

**DOI:** 10.1186/1471-213X-7-77

**Published:** 2007-06-28

**Authors:** Susanna Amadio, Fabrizio Vacca, Alessandro Martorana, Giuseppe Sancesario, Cinzia Volonté

**Affiliations:** 1 Santa Lucia Foundation, Via del Fosso di Fiorano 64, 00143 Roma, Italy; 2 University of Rome Tor Vergata, Facoltà di Medicina, Dipartimento di Neuroscienze, Roma, Italy; 3 Institute of Neurobiology & Molecular Medicine, CNR, Via del Fosso di Fiorano 64, 00143 Roma, Italy

## Abstract

**Background:**

In the CNS, several P2 receptors for extracellular nucleotides are identified on neurons and glial cells to participate to neuron-neuron, glia-glia and glia-neuron communication.

**Results:**

In this work, we describe the cellular and subcellular presence of metabotropic P2Y_1 _receptor in rat cerebellum at two distinct developmental ages, by means of immunofluorescence-confocal and electron microscopy as well as western blotting and direct membrane separation techniques. At postnatal day 21, we find that P2Y_1 _receptor in addition to Purkinje neurons, is abundant on neuronal specializations identified as noradrenergic by anatomical, morphological and biochemical features. P2Y_1 _receptor immunoreactivity colocalizes with dopamine β-hydroxylase, tyrosine hydroxylase, neurofilament light chain, synaptophysin and flotillin, but not with glial fibrillary acidic protein for astrocytes. P2Y_1 _receptor is found enriched in membrane microdomains such as lipid rafts, in cerebellar synaptic vesicles, and is moreover visualized on synaptic varicosities by electron microscopy analysis. When examined at postnatal day 7, P2Y_1 _receptor immunoreactivity is instead predominantly expressed only on Bergmann and astroglial cells, as shown by colocalization with glial fibrillary acidic protein rather then neuronal markers. At this age, we moreover identify that P2Y_1 _receptor-positive Bergmann fibers wrap up doublecortin-positive granule cells stretching along them, while migrating through the cerebellar layers.

**Conclusion:**

Membrane components including purinergic receptors are already known to mediate cellular contact and aggregation in platelets. Our results suggesting a potential role for P2Y_1 _protein in cell junction/communication and development, are totally innovative for the CNS.

## Background

Extracellular nucleotides partake to excitatory neurotransmission and neuromodulation in the CNS and are capable of intervening in a broad array of physiopathological functions acting on different P2 purinergic receptors [1-3]. Because of their heterogeneous faculty of responding to several diverse nucleotides, interest is growing on discovering the exact localization of the various P2 receptor subunits in selected organs, tissues and cellular phenotypes [4,5]. To date, seven different ionotropic P2X (P2X_1–7_) [6] and eight distinct metabotropic P2Y (P2Y_1,2,4,6,11,12,13,14_) [7] receptors were cloned from mammalian species. In contrast to P2X ion channels, P2Y proteins hold the general feature of G protein-coupled receptors with seven hydrophobic transmembrane domains. Since activation of P2Y subtypes leads to second messenger cascades, their response is slower than that mediated by P2X subunits [8-10]. Among P2Y receptors, an elevated expression particularly of the P2Y_1 _subtype was detected in both human [11] and rat brain [12]. In human tissue, expression of P2Y_1 _protein is reported exclusively on neuronal cells in cerebral and cerebellar cortex, in hippocampus, caudate-putamen nuclei, globus pallidus, subthalamic nucleus and midbrain [11]. Moreover, P2Y_1 _protein appears associated to neurofibrillary tangles and neuritic plaques in postmortem brain of Alzheimer's disease patients [13]. On the contrary, P2Y_1 _receptor in rat is detected not only on grey matter, particularly cerebellar, cortical and hippocampal neurons, but also on white matter of corpus callosum and optic nerve [12].

Many studies have already highlighted a general role for purinergic signaling in brain development [14] and in neuron and/or glia function and communication [15-17]. For instance, activation particularly of P2Y_1 _receptor is suggested to regulate oligodendrocyte progenitor functions [18], whereas reduced levels of P2Y_1 _protein apparently affect proliferation and migration, but not differentiation of neural progenitor cells during early CNS development [19]. With the present work, we further study P2Y_1 _receptor and compare protein distribution in juvenile versus neonatal rat cerebellum. We show that during postnatal growth, the phenotypic appearance of P2Y_1 _protein undergoes a drastic switch from glial to neuronal localization, therefore suggesting this receptor as a novel marker of cerebellar development.

## Results

### P2Y_1 _receptor is present on tyrosine hydroxylase- and dopamine β-hydroxylase-positive neurons in juvenile rat cerebellum

**Figure 1:**
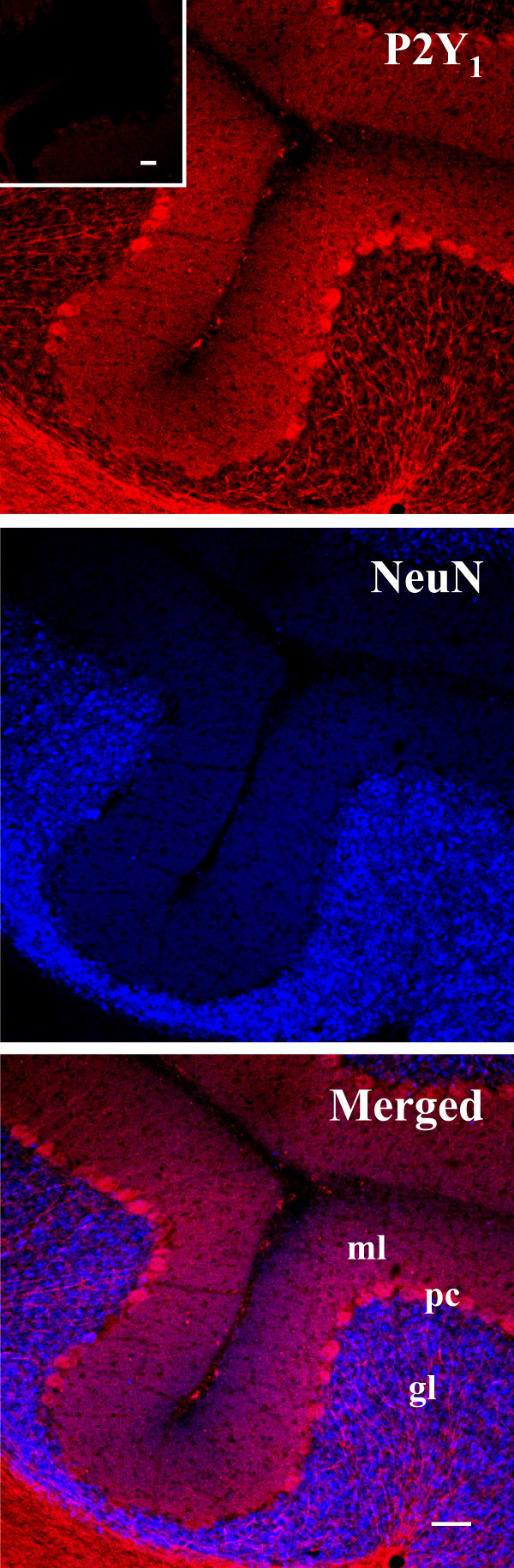
**Map of P2Y_1 _receptor protein in juvenile rat cerebellar cortex**. Sagital sections of P21 rat cerebellar cortex were processed for double immunofluorescence analysis. Confocal images show that P2Y_1 _receptor protein (red immunofluorescence) is present on Purkinje cell (pc) bodies and ramifications, on neuropil of molecular layer (ml), on axons of the white matter irradiating into the granule layer (gl), but not on NeuN-positive granule neurons (blue immunofluorescence). The inset represents immunofluorescence analysis performed in the presence of the immunogenic peptide for P2Y_1 _receptor. Scale bars = 50 μm.

**Figure 2:**
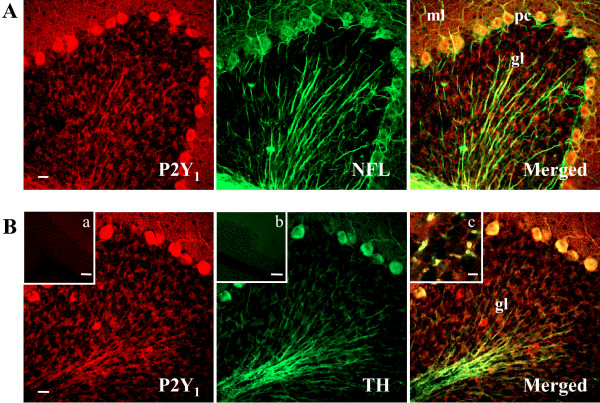
**Localization of P2Y_1 _receptor on neurons**. Confocal images demonstrate that P2Y_1 _receptor immunoreactivity (red Cy3 immunofluorescence) colocalizes with NFL (green Cy2 immunofluorescence) specific for neuronal processes (A) and with TH (green Cy2 immunofluorescence) (B). The insets "a" and "b" in B, represent immunofluorescence analysis performed in the absence of the primary antibodies and only with the anti-rabbit or anti-mouse secondary antibodies, respectively. The inset "c" in B, shows large (1–3 μm diameter) varicosities present on TH-positive fibers expressing P2Y_1 _receptor (yellow immunofluorescence). Abbreviations: ml, molecular layer; pc, Purkinje cells; gl, granule layer. Scale bars are: 20 μm in A and B; 100 μm in the insets "a, b" in B; 5 μm in the inset "c" in B.

**Figure 3:**
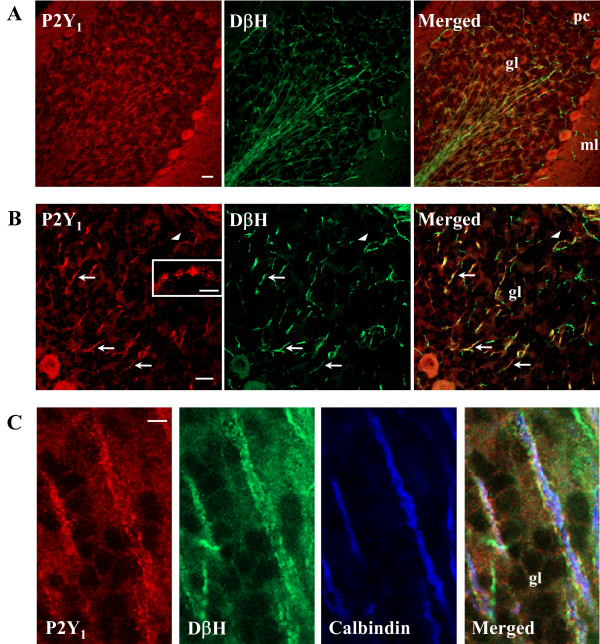
**Localization of P2Y_1 _receptor on DβH neurons**. Confocal images demonstrate that P2Y_1 _receptor immunoreactivity (red Cy3 immunofluorescence) colocalizes with DβH, specific marker of noradrenergic neurons (green Cy2 immunofluorescence). In panel B, a higher magnification of sparse and unbundled noradrenergic fibers of different thickness (arrows) and beaded nature (arrow head and inset) is shown. Panel C represents a triple immunofluorescence performed with anti-P2Y_1 _receptor (red Cy3 immunofluorescence), anti-DβH (green Cy2 immunofluorescence), and anti-Calbindin-D-28K (blue immunofluorescence) antibodies. Scale bars are: 20 μm in A and B, and 5 μm in C and in the inset in B.

**Figure 4:**
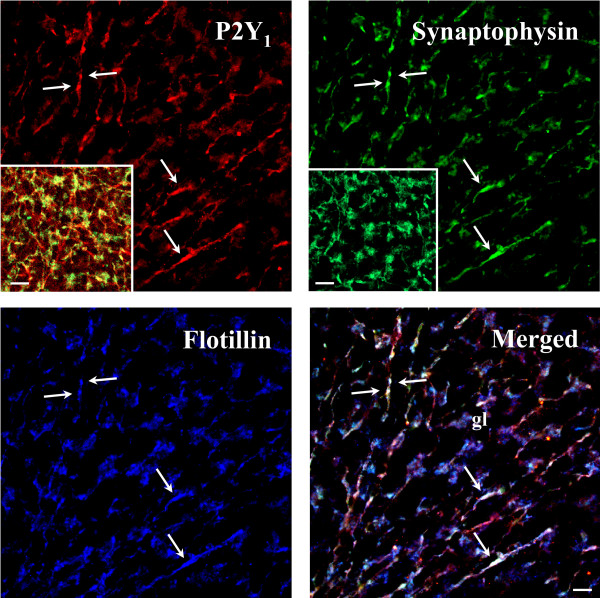
**Beaded appearance of P2Y_1 _receptor immunostaining**. Sagital sections of P21 rat cerebellar cortex were processed for triple immunofluorescence analysis and visualized by confocal microscopy. Rabbit anti-P2Y_1 _(red Cy3 immunofluorescence) was used in combination with mouse anti-synaptophysin, a marker for synaptic structures (green Cy2 immunofluorescence), and mouse anti-flotillin, a lipid rafts-associated integral membrane protein (blue immunofluorescence). In gl, the overlapping immunoreactive signals of P2Y_1_, synaptophysin and flotillin (arrows) can be distinguished by white immunofluorescence (arrows). Scale bars: 10 μm and 20 μm in the insets.

We describe in this work the topographic cellular and subcellular in vivo distribution of P2Y_1 _receptor protein in the cerebellum of juvenile rat at postnatal day 21 (P21). We show by confocal microscopy that P2Y_1 _protein immunoreactivity is uniformly distributed throughout specific zones of cerebellar cortex (Fig. 1). Confirming what was previously depicted by immunohistochemical observations [12], our immunofluorescence studies indicate that P2Y_1 _receptor is limited to Purkinje cell (pc) bodies and ramifications, to neuropil of molecular layer (ml), and to axons of white matter irradiating into granule (gl) and Purkinje (pl) layers (Fig. 1, Fig. 2). In addition, we show that specific P2Y_1 _receptor staining is absent from NeuN-positive granule cell bodies, but present on the conspicuous array of fibers irradiating into the cerebellum and spreading out their projections to granular, Purkinje and molecular layers (Fig. 1). In sagital cerebellar sections, we see by confocal analysis that P2Y_1 _receptor immunoreactivity colocalizes with thick, straight and bundled neurofilament light chain (NFL) fluorescence, specific for neuronal processes (Fig. 2A), with tyrosine hydroxylase (TH), the rate-limiting enzyme in catecholamine biosynthesis (Fig. 2B), and with dopamine β-hydroxylase (DβH), specific for noradrenergic neurons (Fig. 3). We find that, differently from NFL (Fig. 2A), nerve fibers immunoreactive for both TH (Fig. 2B) or DβH (Fig. 3) and P2Y_1 _receptor can be much thinner (Fig. 3B, arrows), sparse, unbundled and winding and, moreover, display abundant varicosities and beaded appearance (Fig. 2B, inset c; Fig. 3B, arrow head and inset). Although P2Y_1 _receptor labeling is not uniformly distributed along the entire fiber, and not all DβH ramifications are P2Y_1 _positive, P2Y_1 _fibers are densely detected in the white matter of each cerebellar folium without distinction among lobules, sometimes in contact with perikarya (data not shown). At higher magnification and by triple immunofluorescence confocal analysis, we observe that a blue Calbindin-positive Purkinje axon is surrounded by yellow colocalizing signals of red P2Y_1 _receptor and green DβH (Fig. 3C). In this particular fiber, the P2Y_1 _receptor is absent from the Purkinje axon, but enriched on noradrenergic terminals on the same axon. Moreover, the numerous and large varicosities (1–3 μm diameter) of TH-positive fibers [20] directly express P2Y_1 _receptor (Fig. 2B, inset c) and are, therefore, likely responsible for the beaded appearance of P2Y_1 _receptor immunostaining. By triple immunofluorescence and confocal analysis on histological sections, we further prove that the P2Y_1_receptor-positive varicosities (red immunofluorescence) are enriched in synaptophysin (specific marker of synaptic structures, green immunofluorescence), and flotillin (a lipid rafts-associated integral membrane protein, blue immunofluorescence) [21], therefore providing a completely overlapping P2Y_1_-synaptophysin-flotillin immunoreactive signal (white immunofluorescence, Fig. 4). The large oval/cuboid staining (Fig. 4 insets), as well as the fine punctate staining, further demonstrate the presence of synaptophysin and P2Y_1 _receptor labeling on synaptic glomeruli.

On the other hand, P2Y_1 _receptor does not colocalize with glial fibrillary acidic protein (GFAP), therefore excluding its concurrent expression on astrocytes (Fig. 8B, lower-right inset).

### Ultrastructural analysis indicates that P2Y_1 _receptor is present on both axon terminals and dendrites

**Figure 5:**
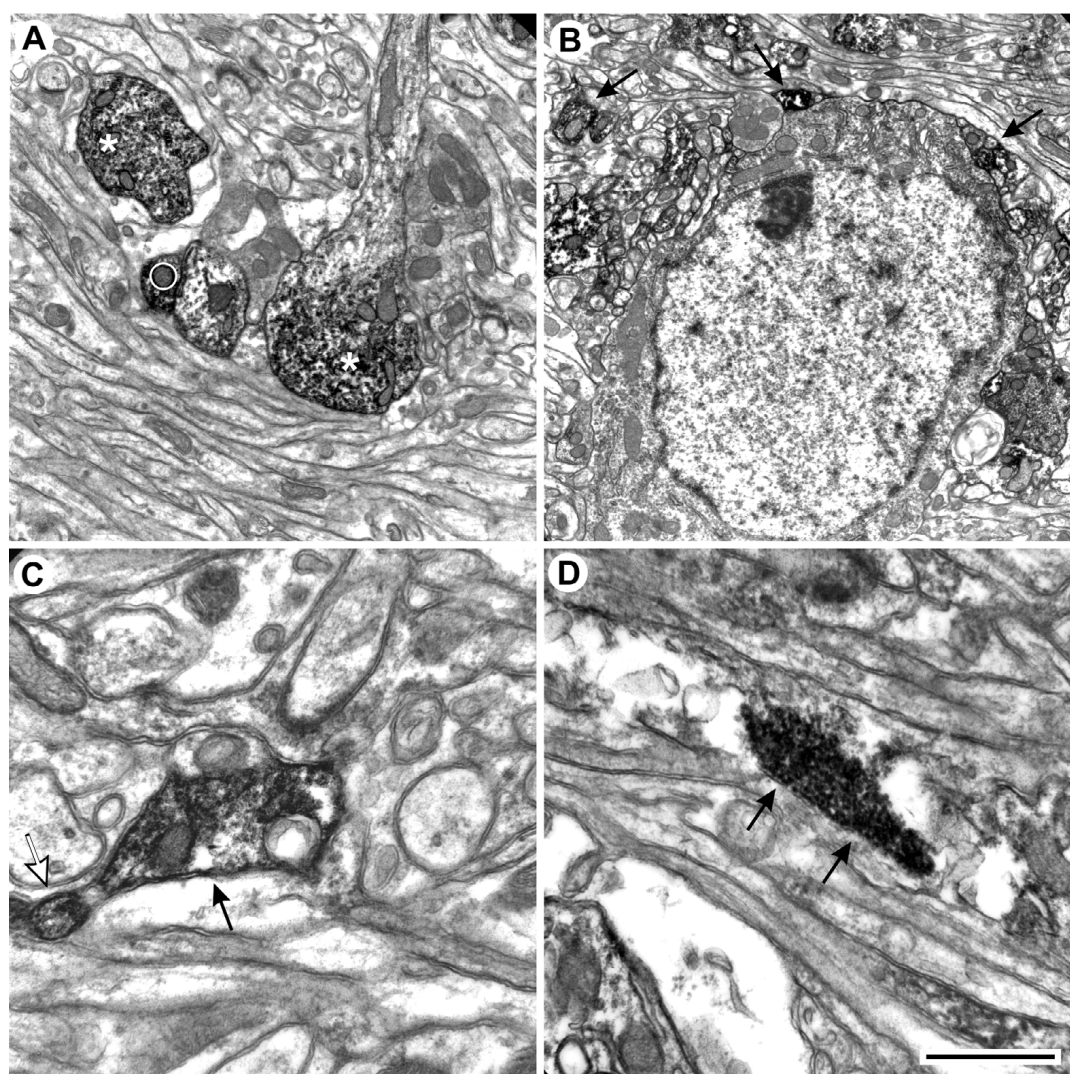
**Electron microscopy analysis of P2Y_1 _receptor on both axon terminals and dendrites**. In A, P2Y_1 _receptor immunolabelling is shown on varicosities of fibers (white circle) and dendrites of granule cells (white asterisks). In B, P2Y_1 _receptor immunolabelled varicosities (black arrows) are shown apposed to neuronal elements such as dendrites and cell bodies. In C, P2Y_1 _receptor immunolabelled varicosity (black arrow) contains mitochondria and pleomorphic vesicles, whereas the intervaricose segment (white arrow) is deprived of vesicles. In D, the varicosity is shown to be filled with pleomorphic vesicles, without forming a clear synaptic contact. Scale bars = 2 μm in A; 2,5 μm in B; 1 μm in C and D.

**Figure 6:**
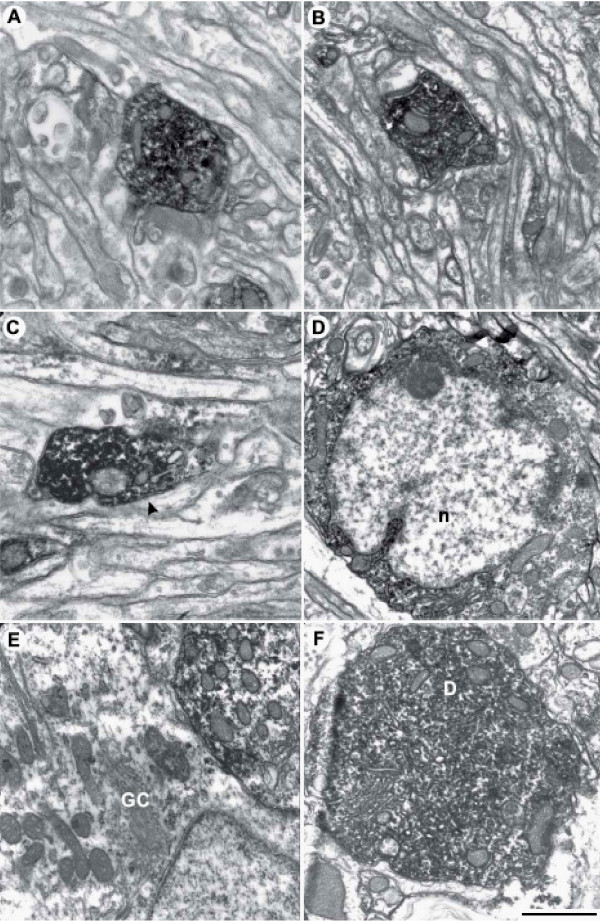
**Electron microscopy analysis of P2Y_1 _receptor on synaptic terminals forming varicosities**. In A, labelled varicosities are apposed to neuronal elements of cerebellar cortical layers, mostly dendrites in granule cell layer. In a few cases, the immunoreactivity is so strong to cover the vesicles of the terminal. In B, the features of the varicosities are unchanged also in Purkinje cell layer. In C, the absence of synaptic junctions is clearly visible (see arrow). In D, the granule cell shows specific labelling in the cytoplasm (n = nucleus of granule cell). In E, a few granule cells (GC) are labeled, being on the contrary apposed to immunolabeled terminals. In F, also Purkinje neurons are immunolabeled, especially proximal dendrites (D). Scale bar in A-C = 1 μm; in D = 1,2 μm; in E and F = 0,8 μm.

To further investigate the subcellular localization of P2Y_1 _protein, we performed ultrastructural analysis by electron microscopy. We confirm the presence of P2Y_1 _receptor immunoreactive profiles in all cortical layers of juvenile rat cerebellar cortex, although more abundantly in gl (Fig. 5A), rather than pl or ml (Fig. 5B). P2Y_1 _receptor immunoreactivity is moreover present on both dendrites (Fig. 5A, asterisks) and fine, beaded axons (Fig. 5A, white circle, Fig. 5B, arrows) that travel along the cerebellar cortical layers. P2Y_1 _immunoreactive axons are un-myelinated and consist of P2Y_1_-positive small axonal enlargements or varicosities (Fig. 5C, black arrow) connected by narrower P2Y_1_-positive inter-varicose segments (Fig. 5C, white arrow). The varicosities are about 1–2 μm in diameter and filled with synaptic pleomorphic vesicles and mitochondria (Fig. 5C and 5D). Intervaricose segments do not contain vesicles. The most remarkable feature of the electron microscopy analysis is the absence of visible synaptic specializations (Fig. 6A–C). The P2Y_1 _receptor-positive varicosities are in fact adjacent to cerebellar elements like granule dendrites (Fig. 6A–C) and cell bodies (Fig. 6E), without formation of conventional synaptic junctions (see arrow in Fig. 6C). In addition, specific immunolabelling is observed also in proximal dendrites of Purkinje neurons (Fig. 6F), and very rarely in cell bodies of granule cells (Fig. 6D). No P2Y_1 _receptor-immunolabelled glial cells are observed.

### P2Y_1 _receptor localizes in lipid rafts and synaptosomes

**Figure 7:**
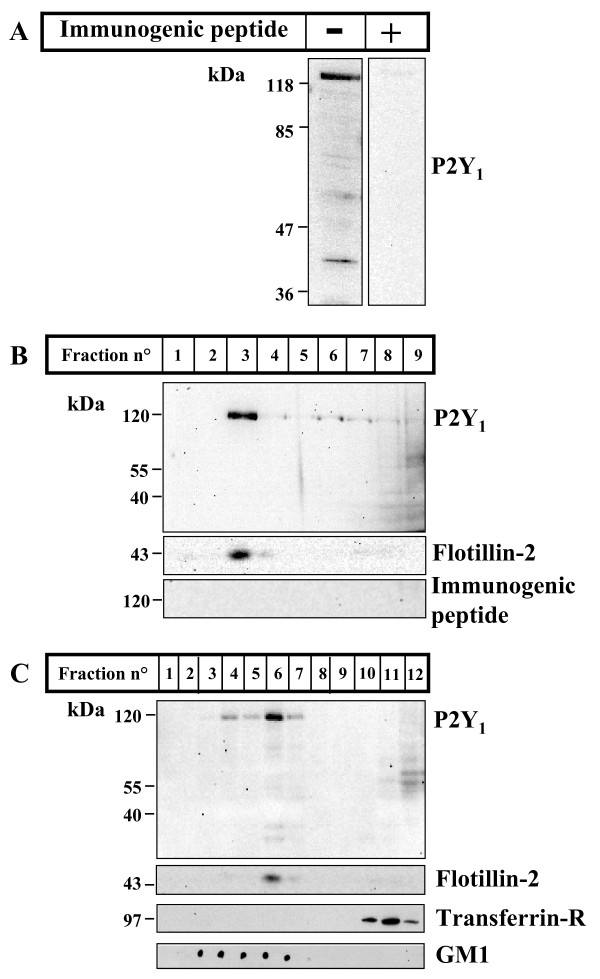
**P2Y_1 _receptor localizes in lipid rafts**. Cerebella were removed from P21 rats and cell protein extracted in lysis buffer for 30 min on ice. Supernatants obtained after a centrifugation for 10 min at 15000 × g were loaded on SDS-PAGE (100 μg/lane), transferred to nitrocellulose and probed with anti-P2Y_1 _receptor antiserum, in the absence (-) or presence (+) of the immunogenic peptide (A). Lipid rafts-enriched fractions were then prepared from total cerebellar tissue (B) or from purified cerebellar synaptosomes (C), both obtained from P21 rats. P2Y_1 _receptor is recovered in rafts fractions 3 (B), in the absence of the immunogenic peptide, or 4–7 (C), together with the selective lipid rafts markers flotillin-2 (B, C) or GM1 (C), but not the transferrin receptor (C), a protein known to be excluded from lipid microdomains.

Since fluorescence microscopy indicated a high degree of co-localization between P2Y_1 _receptor and the lipid rafts marker flotillin-2, we purified low buoyant density detergent-resistant membranes (lipid rafts) from P21 rat cerebellum, in order to better dissect the localization of P2Y_1 _protein. Whereas in total cerebellar extracts the P2Y_1 _receptor is recognized as a major protein band of approximately 120 kDa and a minor band of about 42 kDa (molecular mass expected from aminoacid sequence analysis) (Fig. 7A), the specific form recognized in the absence of the antigenic peptide by the P2Y_1 _receptor antiserum in the low density fractions of the sucrose gradient (together with the lipid rafts marker flotillin-2) corresponds not to the monomeric, but to the oligomeric protein (Fig. 7B). This is not surprising, since SDS-resistant oligomeric forms of this receptor are frequently reported, for instance in vascular smooth muscle and endothelial cells, and suggested to be the functional forms [22]. Consistently, when the receptor antiserum used to identify P2Y_1 _protein is pre-incubated with the immunizing peptide, the reactivity not only to the monomeric, but also to the oligomeric band is completely abolished (Fig. 7A,B), therefore confirming the specificity of the antigen-antiserum interactions. This is further proved by the result that the P2Y_1 _receptor polyclonal antiserum used in this study is able to efficiently immunoprecipitate the recombinant Myc-P2Y_1 _human receptor transiently transfected in SH-SY5Y cells and detected by the anti-myc 9E10 antibody (data not shown).

Since fluorescence microscopy also demonstrated a high degree of co-localization between P2Y_1 _receptor and synaptophysin (Fig. 4), fractions were also prepared directly from cerebellar synaptosomes, in order to confirm the synaptic localization of lipid rafts-resident P2Y_1 _receptor. Our results again demonstrate that only the P2Y_1 _receptor oligomer is present in synaptosomal lipid rafts-enriched fractions (Fig 7C), together with the lipid rafts markers flotillin-2 and GM1ganglioside (fractions 4–7). Neither monomer nor oligomer receptor is instead detected in the high density synaptosomal fractions (fractions 10–12) containing transferrin receptor, a protein known to be excluded from lipid rafts microdomains.

### The phenotypic expression of P2Y_1 _receptor switches during cerebellar development

**Figure 8:**
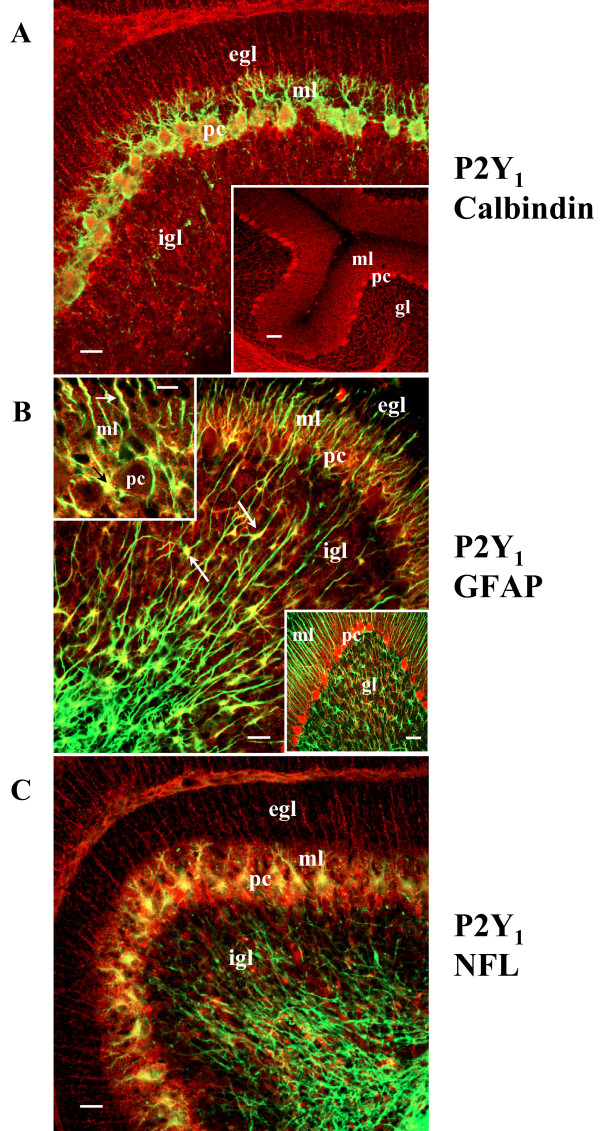
**Map of P2Y_1 _receptor protein in neonatal versus juvenile rat cerebellar cortex**. Double immunofluorescence visualized by confocal analysis was performed in neonatal rat (P7) cerebellar cortex and compared to that at P21 (inset in panel A). At P7, P2Y_1 _receptor immunoreactivity (red Cy3 immunofluorescence) is present on calbindin-positive pc (green Cy2 immunofluorescence), on fibers irradiating from the thin ml toward the egl (A, B), on cell bodies at the interface between the pl and ml (black arrow in the left inset of panel B), and on isolated cells situated in the igl (white arrows in panel B). Double immunofluorescence (yellow) shows complete colocalization between P2Y_1 _(red Cy3 immunofluorescence) and GFAP (green Cy2 immunofluorescence) signals (white arrows in B; black and white arrows in the upper-left inset of panel B), differently from what observed at P21 (lower-right inset of panel B). In C, P2Y_1 _(red Cy3 immunofluorescence) and NFL (green Cy2 immunofluorescence) immunostaining are shown. Abbreviations: egl, external granule layer; gl, granule layer; igl, internal granule layer; ml, molecular layer; pc, Purkinje cells. Scale bar = 20 μm in A, B and C; 50 μm in the inset in A; 10 μm in the upper-left inset in B; 40 μm in the lower-right inset in B.

We next investigated the expression of P2Y_1 _receptor at postnatal day 7 (P7), when the maturation of synapses is just taking place between granule neuron dendrites and fiber terminals [23], and the internal granular layer (igl) rapidly expands beneath the monocellular sheet of pc. With the only exception of calbindin-positive pc (Fig. 8A), we find that P2Y_1 _receptor localization is completely different from that observed at P21 (Fig. 8A, inset). At P7, P2Y_1 _receptor immunoreactivity is indeed absent from noradrenergic fibers of white matter, but predominantly shown: a) on fibers irradiating the thin ml (above the pl) and the external granule (or germinal) layers (egl) (Fig. 8B); b) on cell bodies aligned at the interface between the pc and ml (Fig. 8B, upper-left inset); c) on isolated cells located in the igl (Fig. 8B). P2Y_1 _receptor immunostaining is thus totally absent from NFL-positive (Fig. 8C) and TH- or DβH-positive fibers (data not shown), which already span into the gl at this age of development. By means of distinctive morphology of cerebellar astrocytes and positive colocalization between P2Y_1 _receptor and GFAP (Fig. 8B), at P7 we do identify P2Y_1 _receptor on both Bergmann cell bodies/fibers and astroglial cells scattered among granule neurons. This is confirmed for postnatal day one (data not shown), but is the opposite of what was shown at P21, when P2Y_1 _receptor is totally absent from GFAP-positive structures (Fig. 8B, lower-right inset) and present only on neuronal fibers (Figs. 2, 3, 4). Conversely, the enrichment of P2Y_1 _receptor in lipid rafts, but not synaptosomal fractions, is confirmed also at P7 (data not shown), therefore appearing as a conserved feature of this receptor.

**Figure 9:**
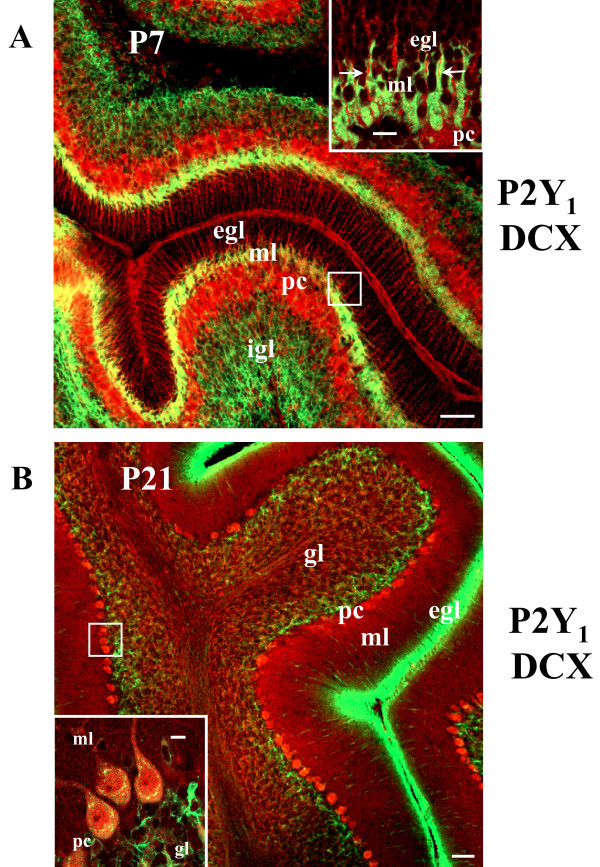
**P2Y_1 _receptor and migrating granule neurons**. Confocal images of DCX (green Cy2 immunofluorescence) and P2Y_1 _(red Cy3 immunofluorescence, white arrows in the inset of panel A) are shown from cerebellar cortex of neonatal rat at P7 (A) and P21 (B). Abbreviations: egl, external granule layer; gl, granule layer; igl, internal granule layer; ml, molecular layer; pc, Purkinje cells. Scale bar = 50 μm in A and B; 10 μm, in the inset in A and B.

It's well known that, during development, Bergmann fibers are associated with migrating granule cells, from which is derived the concept of glia-guided neuronal migration [24], and that DCX is a distinctive marker of granule cells only during the period of radial descent into the deep cerebellar layers, when it directs neuronal movement through the organization and stability of microtubules [25]. Therefore, we used DCX immunoreactivity to observe the potential relationship between Bergmann glia (P2Y_1_-positive only at neonatal ages P1–P7) and granule neurons. We find that at P7, the DCX-positive signal appears as a diffuse labeling on both the entire igl and the thin ml enriched in radially migrating granule cells, but is excluded by the pre-migratory P2Y_1_-expressing egl (Fig. 9A). Given the high density, amoeboid shape and lack of defined neuronal contour of migrating granule cells at this neonatal age, the DCX-positive staining appears contiguous to P2Y_1_-GFAP fibers (Fig. 9A inset), providing an almost overlapping signal when observed at low magnification (Fig. 9A, yellow fluorescence). Nevertheless, at higher resolution it is possible to distinguish that DCX-positive granule cells are wrapped up and stretched along P2Y_1_-GFAP-positive Bergmann fibers (Fig. 9A inset). On the opposite, when at P21 the egl almost disappears after complete proliferation and migration of granule neurons [23], the DCX immunostaining is confined to a very narrow zone below the pc (Figs. 9B, inset) and P2Y_1 _receptor is no longer detected on Bergmann glia (Fig. 8B lower-right inset and Fig. 9B).

## Discussion

The main goal of our study was to characterize the cellular and subcellular distribution and features of the purinergic metabotropic P2Y_1 _receptor in the cerebellum, and to gain insights on its potential function during development of the cerebellar circuitry. To this purpose, we analyzed P2Y_1 _receptor expression at P21, when the major structural changes already took place in the developing cerebellum, and compared it with expression at P7, when migration of granule neurons from the molecular to the granular layer is still occurring [23,26,27], and when the neuronal bodies in the locus coeruleus of origin for the dorsal bundle of noradrenergic neurons innervating the cerebellum are spreading out their projections to the granular, Purkinje and molecular layers [28]. In juvenile rat cerebellum we find that, in addition to Purkinje cell bodies and ramifications [12], P2Y_1 _receptor is abundant on neuronal specializations identified as noradrenergic by anatomical (the fibers travel in linear, sparse and different thickness profiles through the gl, and branch into radially and longitudinally oriented chains), ultrastructural (abundance of synaptic varicosities and winding, beaded appearance) and biochemical features (immunoreactivity with noradrenergic-specific DβH absent from Purkinje neurons in rat) [29]. Moreover, we observe that P2Y_1 _receptor is preferentially localized on synaptic varicosities of the noradrenergic fibers and synaptic glomeruli, which can be recognized by the presence of synaptophysin. The ultrastructural analysis confirms the localization of P2Y_1 _receptor on varicosities of axon terminals, moreover showing features indicative of a particular modality of transmitter release. As generally reported for noradrenergic fibers [30], the absence of conventional synaptic junctions led us to suppose that in the cerebellum also extracellular nucleotides might exert their modulatory action through paracrine release or simple diffusion in the extracellular space. This modality has the advantage to extend the potential effects of extracellular nucleotides, and neurotransmitters in general, to structures equipped with adequate receptors (extra-synaptic receptors) [31], but distant from the source of release (volume transmission) [32]. The enrichment in these P2Y_1_-positive varicosities of both synaptophysin and the lipid rafts marker flotillin-2 (together with the biochemical observation that P2Y_1 _receptor is indeed a lipid rafts-resident protein in total cerebellar tissue and cerebellar synaptosomes) furthermore suggests that the role of P2Y_1 _receptor is likely mediated by lipid microdomains. In this regard, it is already well-known that lipid rafts display a key part in the targeting and functional organization of proteins at both synapses and spines [33].

Since a further specification of the central noradrenergic system consists in the frequency of co-transmission phenomena, the presence in the cerebellum of P2Y_1 _receptor on noradrenergic neurons in addition to Purkinje cells might signify that extracellular nucleotides such as the natural agonists ATP and ADP could contribute in this brain region to the physiological role of noradrenaline [34]. In this regard, ATP is already well known to be co-released with noradrenaline in various PNS and CNS neurons [35,36]. Thus, if co-release and synergism occur at cerebellar synapses as well, nucleotide-dependent increase of cerebellar noradrenergic signaling could be achieved. Homologous recombination techniques have shown that complete removal of either the enzymes responsible for noradrenaline metabolism or the vesicular monoamine transporter has deleterious consequences for foetal survival [37]. Moreover, blockade of postsynaptic noradrenergic receptors decreases the rate of learning in several cerebellar-dependent motor tasks [38]. The possibility of integrating and complementing noradrenergic with purinergic mechanisms to increase the strength of synaptic connections through activation of both noradrenergic and P2Y_1 _receptors might therefore constitute also a novel powerful approach against neuronal degeneration and/or malfunctioning in the cerebellum.

The presence of P2Y_1 _receptor in postnatal rat cerebellum was then analyzed before the formation of noradrenergic synaptic connections takes place. Surprisingly, we have shown here that P2Y_1 _undergoes a drastic switch during development, with a phenotypic expression resembling that of Bergmann glia at P7, rather then noradrenergic neurons at P21. In this regard, it is well established that a number of neuronal and glial receptor systems and/or diffusible factors act to induce and maintain Bergmann glia process extension at an early stage of postnatal development [39-41], when Bergmann cells specialize in supporting the migration of granule neurons and migration of granule cells seems to be largely dependent on their interaction with glial processes [42,43]. In particular, the ErbB4 tyrosine kinase receptor present on Bergmann glia appears to have a distinct role in this process [44], interacting with neoregulin expressed on migrating granule cells [45,46]. On the other hand, in vitro studies on the migration of granule neurons demonstrated that the glycoprotein astrotactin provides a neuronal receptor system for migration along glial processes [47]. Thus, P2Y_1 _receptor expression only on Bergmann glia at an early stage of development might sustain a role for purinergic receptors in signaling events needed for interaction and migration of neurons. This is strongly supported by the result that ADP, preferential ligand for P2Y_1_receptor, induces Ca^2+ ^mobilization in Bergman glia [48]. The factors produced by granule neurons to induce the glial scaffold might thus comprise the purinergic ligands ATP and ADP directly targeting P2Y_1 _receptors. To support this hypothesis, we have both shown here that P2Y_1 _receptor is localized at the interface between Bergmann glia and DCX-positive migrating granule cells, and previously demonstrated that, at least in vitro, granule neurons can release ATP, which is easily degraded to ADP in the extracellular environment [49]. It is finally well known that, once migration across the glia scaffold is completed and cells change their repertoire of adhesive molecules and switch into a static asset: a) the Bergmann glia loses the apposition with granule neurons; b) the granule cells are locked in position by the formation of new specific axon-target interactions [46]. In parallel, we have demonstrated here that in juvenile rat cerebellum: a) the Bergmann glia lose P2Y_1 _receptors; b) P2Y_1 _receptors appear on Purkinje and noradrenergic neurons forming new specific axon-target interactions.

## Conclusion

Since membrane components of the cell surface are largely known to mediate the close apposition between two cells during all phases of development, our results suggest a novel role for P2Y_1 _receptor in the CNS, specifically in cell junction/communication. This already occurs on platelets, where P2Y_1 _receptor is well known to mediate cell contact and aggregation [50-52]. Considering the glia-neuron switch of P2Y_1 _receptor a novel biological mechanism of development, aim of further studies will be to investigate the potential impact of this receptor on Bergman glia-guided migration of granule neurons; on alteration/reorganization of noradrenergic fibers in the cerebellar cortex in response to the degeneration of their major target, granule and Purkinje neurons; and finally to investigate if the presence of purinergic ligands in the environment surrounding noradrenergic fibers can influence their anatomical integrity and development.

## Methods

### Histological procedures

Wistar rats (Harlan, Udine, Italy) of different ages were deeply anesthetized by i.p. injections of sodium pentobarbital (60 mg/kg), and transcardially perfused with saline (0.9% NaCl) followed by 4% paraformaldehyde, in phosphate buffer (PB, 0.1 M pH 7.4). Each brain was immediately removed, post-fixed in the same fixative for 2 hs, and then transferred to 30% sucrose in PB at 4°C, until it sank. The experimental protocol used in this study was approved by the Italian Ministry of Health and was in agreement with the guidelines of the European Communities Council Directive of November 24, 1986 (86/609/EEC) for the care and use of laboratory animals. All efforts were made to minimize the number of animals used and their suffering.

### Double immunofluorescence

Sagital sections (40 μm thick) were cut on a freezing microtome and were processed for double immunofluorescence studies. Non-specific binding sites were blocked with 10% normal donkey serum in 0.3% Triton X-100, in phosphate buffered saline (PBS) for 30 min at room temperature. The sections were incubated in a mixture of primary antisera for 24 hs in 0.3% Triton X-100 in PBS. Rabbit anti-P2Y_1 _(1:500, Alomone, Jerusalem-Israel) was used in combination with either mouse anti-Calbindin-D-28K (1:200, Sigma, Mi-Italy), mouse anti-Tyrosine Hydroxylase (TH, 1:500, Sigma), mouse anti-Dopamine β-Hydroxylase (DβH, 1:500, Chemicon International, Inc. Temecula, CA-USA), mouse anti-Glial Fibrillary Acidic Protein (GFAP) (1:400, Sigma), mouse anti-Synaptophysin (1:100, Sigma), goat anti-Doublecortin (DCX, 1:200, Santa Cruz, Mi-Italy), or goat anti-NFL (Neurofilament-L protein, 1:100, Santa Cruz). The secondary antibodies used for double labeling were Cy3-conjugated donkey anti-rabbit IgG (1:100, Jackson Immunoresearch, West Baltimore Pike, PA, USA, red immunofluorescence), Cy2-conjugated donkey anti-mouse IgG (1:100, Jackson Immunoresearch, green immunofluorescence) or Cy2-conjugated donkey anti-goat IgG (1:100, Jackson Immunoresearch, green immunofluorescence). The sections were washed in PBS three times for five min each, and then incubated for 3 hs in a solution containing a mixture of the secondary antibodies in 1% normal donkey serum in PBS. After rinsing, the sections were mounted on slide glasses, allowed to air dry and coverslipped with gel/mount™ anti-fading medium (Biomeda, Foster City, CA-USA).

### Triple immunofluorescence

After double immunofluorescence, the sections were mounted on slide glasses, and allowed to air dry. A rectangle was then drawn around the sections with a PAP pen. To allow the use of a second mouse antibody in the same immunolabeling protocol, the unlabeled monoclonal anti-NeuN (Neuronal Nuclei, mouse IgG_1 _isotype or anti-Calbindin-D-28K (mouse IgG_1 _isotype)) or Flotillin-2 (mouse IgG_1 _isotype) were labeled with Zenon technology (Molecular Probes, Oregon, USA). Briefly, mouse anti-NeuN (1:100, Chemicon International), mouse anti-Flotillin-2 (1:100, BD Biosciences, San José, CA) and mouse anti-Calbindin-D-28K (1:200, Sigma) were separately incubated with Zenon Alexa Fluor 647 mouse IgG_1 _labeling reagent (molar ratio 6:1), which contains a fluorophore-labeled (Ex/Em 650/668) anti-mouse Fab fragments. The labeled Fab fragments bind to the Fc portion of the monoclonal antibodies and excess Fab fragments are neutralized by the addition of a nonspecific IgG (Zenon blocking reagent-mouse IgG). The addition of non-specific IgG prevents cross-labeling of the Fab fragment, in experiments where multiple primary antibodies of the same type are present. After rehydration in PBS, the sections were incubated with the staining solution in PBS containing 0.5% Triton X-100 (PBT) in a humidified chamber for 2 hs at room temperature. The sections were washed twice in PBT and for 5 min in PBS at room temperature. Sections were then fixed in 4% paraformaldehyde in PB for 15 min at room temperature, to avoid the dissociation of the Zenon Fab fragment from the primary antibody, washed three times with PBS, allowed to air dry and coverslipped with gel/mount anti-fading medium.

### Confocal microscopy

Double or triple label immunofluorescence was analyzed by means of a confocal laser scanning microscope (CLSM) (LSM 510, Zeiss, Arese Mi-Italy) equipped with argon laser emitting at 488 nm, helium/neon laser emitting at 543 nm, and helium/neon laser emitting at 633 nm. Specificity of the antibodies was positively proved by performing confocal analysis in the absence of the primary antibodies, but in the presence of either anti-rabbit or anti-mouse secondary antibodies. Specificity was further confirmed for the P2Y_1 _antiserum by performing immunoreactions in the simultaneus presence of the P2Y_1 _neutralizing immunogenic peptide.

### Electron microscopy sample preparation

Rats (n = 4) were anaesthetised with chloral hydrate (400 mg/kg i.p.), perfused through the ascending aorta with a solution of NaCl 0.9% for 5 min, then followed by 3% paraformaldehyde with 0.4% glutaraldehyde in PB for 30 min. Cerebella were dissected and sagittal sections were cut at 40 μm, and washed several times in PBS. Sections were treated with sodium borohydrate (Sigma) 0.1% in PBS. Immunoreactivity for P2Y_1 _receptor was detected by means of the avidin-biotin peroxidase method. Briefly, sections from the cerebellum were pre-blocked in a solution containing 10% goat serum in PB for 30 min at room temperature. Then, sections were incubated in a solution containing primary antibody against P2Y_1 _receptor (rabbit anti-P2Y_1_diluted 1:200) in PB for 24 hs at 4°C. After several washes in PBS, sections were incubated with biotinylated secondary antibody (goat anti-rabbit diluted 1:100 Vectastain Elite, Vector Laboratories, Peterborough, UK) in PB for 3 hs at room temperature. They were then incubated in avidin-biotin peroxidase complex (diluted 1:100 in PB; Vectastain Elite, Vector Labs.) for 1 h. After washing, immunolabeling was revealed by incubation of the sections in 0.05% 3,3'-diaminobenzidine solution (DAB-Sigma) diluted in Tris-HCl buffer, in the presence of 0.01% H_2_O_2_. The reaction was stopped by several washes in Tris-HCl buffer followed by PBS. Sections were post-fixed in osmium tetroxide (1% in PB) for 10 min, dehydrated in ascending series of dilution of ethanol (with the presence of 1% uranyl acetate in 70% ethanol) followed by propylene oxide (Aldrich, MI, Italy) and then embedded overnight in resin (Durcupan ACM-Fluka, Gillingham, Dorset, UK), mounted on glass slides and then cured at 60°C for 48 hs. The areas of interest were examined in the light microscope, cut from the sections and 60 nm ultra-thin sections were obtained with an ultramicrotome (Reichert-Jung Ultracut E, Leica, Nussloch, Germany), and collected on 400-mesh copper grids, counterstained with lead citrate and examined using a Zeiss EM900 electron microscope. Controls were performed omitting the primary antibody from the procedure.

### Electron microscopy data analysis

The ultrastructural analysis was performed exclusively on the most superficial portions of the tissue in contact with the embedding plastic, in order to minimize artificial differences in labeling attributed to potential differences in the penetration reagents. Regions used for this analysis were chosen on the basis of P2Y_1 _receptor immunoreactivity and the morphological integrity of the tissue. The labelled profiles were examined in thirty-two ultra-thin sections from three separate rats, in four sections each taken from the vermis, the cerebellar hemispheres and the cerebellar nuclei. Electron micrographs of immunoreactive structures were taken at magnifications of 7000–30000 X, then printed and used as the sampling region of each block. The classification of neuronal elements was made according to the description of Peters et al., [53]. Neuronal somata were identified by the nucleus, Golgi apparatus, and rough endoplasmic reticulum; un-myelinated axons were distinguished from dendrites by their larger diameter and/or the abundance of uniformly distributed microtubules and synaptic inputs from axon terminals. Neuronal profiles were classified as un-myelinated axons if they were 0.1–0.25 μm in cross sectional diameter and contained microtubules and/or small vescicles. Axon terminals were defined as elements 0.25 μm or larger in diameter containing numerous small synaptic vescicles. Synapses formed by axon terminals were defined as asymmetric, when their post-synaptic density was thicker that the pre-synaptic one, and as symmetric, when both membranes showed equal electron density. Two structures were considered adjacent, when the two plasma membranes were parallel and not separated by glial processes, but no membrane specialization was visible.

### Preparation of low density membrane fractions

Detergent-insoluble glycolipid fractions were prepared following an established method [54], with minor modifications [55]. Briefly, cerebella were removed from 21 days old Wistar rats, cut with a M_c_Ilwain tissue chopper (400 μm) in two orthogonal directions and resuspended in 10 volumes (w/v) of ice-cold lysis buffer (10 mM Tris-HCl pH 7.5, 150 mM NaCl, 1% Triton X-100, 2 mM EDTA, 1 mM PMSF, 20 μM Leupeptin) by vortexing. After 30 min, the lysate was centrifuged at 2000 × g for 10 min, to remove nuclei and large debris. The resulting supernatant (500 μl, 2–2.5 mg) was mixed with an equal volume of 85% sucrose in TBS (10 mM Tris-HCl pH 7.5, 150 mM NaCl), and placed at the bottom of the centrifuge tube. A volume of 700 μl of 35% sucrose in TBS and 400 μl of 5% sucrose were layered on the top of the lysate. The gradient was centrifuged for 14 hs at 200.000 × g in a TLS 55 rotor (Beckman Instruments, Porterville, CA). Four fractions of 150 μl and five fractions of 300 μl were collected from the top of the tube. The entire procedure was performed at 4°C. Equal volumes, containing a range of approximately 0.5–100 μg of total protein depending on the fraction, were loaded on SDS-PAGE electrophoresis.

### Synaptosome Triton X-100 solubilization and sucrose floatation gradients

Synaptosomes were obtained from rat cerebella by means of differential centrifugation, as previously described [56]. Briefly, post-nuclear supernatants were centrifuged at 9200 × g for 15 min to yield a pellet corresponding to partially purified synaptosomes. These pellets (6 mg of proteins) were resuspended in 0.75 ml of buffer A (150 mM NaCl, 2 mM EGTA, 50 mM Tris-HCl, pH 7.5, protease inhibitors) containing 1% (w/v) Triton X-100. After 30 min on ice, each sample was adjusted to 1.2 M sucrose, placed in a centrifuge tube and overlaid with a linear gradient ranging from 30 to 5% sucrose (all prepared in buffer A). The gradients were centrifuged at 190000 × g for 19 hs using a rotor SW 41 Ti (Beckman Instruments). Fifteen fractions (0.8 ml each), and the pellets resuspended in 0.8 ml of buffer A were collected and analysed by means of SDS-PAGE and western blotting. The sucrose concentration in each fraction was determined by refractometry.

### Western blot analysis

Equal amount of sucrose gradient samples was separated by electrophoresis on 10%–12% SDS-PAGE and transferred to nitrocellulose membranes Hybond-C extra (Amersham Biosciences, Cologno Monzese, Italy). The filters were pre-wetted in 5% non-fat milk in TBS-T (10 mM Tris pH 8, 150 mM NaCl, 0.1%Tween 20), hybridized overnight with rabbit anti-P2Y_1 _(1:400) and with mouse anti-Flotillin-2 (1:1000), followed by horseradish peroxidase-coupled secondary antibody, and analysed by ECL chemiluminescence (Amersham Biosciences), using Kodak Image Station (KDS IS440CF).

### Anti-P2Y_1 _specificity

The polyclonal P2Y_1 _antiserum used in this study was raised against a P2Y_1 _receptor highly purified peptide (identity confirmed by mass spectroghraphy and aminoacid analysis), corresponding to a specific epitope not present in any other known protein: residues 242–258 of rat and human P2Y_1 _(3^rd ^intracellular loop). The specificity of the P2Y_1 _receptor signal was assessed by incubating western blots either in the absence of the primary antiserum, or in the presence of the primary antiserum together with the neutralizing P2Y_1 _immunogenic peptide (μg protein ratio 1:1 between peptide and antiserum). Furthermore, the P2Y_1 _receptor polyclonal antiserum was proved to efficiently immunoprecipitate the recombinant Myc-P2Y_1 _human receptor transiently transfected in SH-SY5Y cells.

## Abbreviations

DCX, doublecortin; DβH, dopamine β-hydroxylase; egl, external granule layer; GFAP, glial fibrillary acidic protein; gl, granule layer; igl, internal granule layer; ml, molecular layer; NeuN, neuronal nuclei; NFL, neurofilament-L protein; pc, Purkinje cells; pl, Purkinje layer; postnatal day 7, P7; postnatal day 21, P21; TH, tyrosine hydroxylase.

## Authors' contributions

SA carried out the immunohistological analysis, participated in the western blot examination and in the design of the study. FV purified lipid rafts from total cerebellar tissue and participated in the western blot examination. AM and GS performed electron microscopy analysis. CV conceived and coordinated the study, and participated in its design. All authors read and approved the final manuscript.
